# Pelvic Abscess with Gangrene of the Vermiform Process

**Published:** 1893-09

**Authors:** A. H. Schenck

**Affiliations:** Kenney, Texas


For Texas Medical Journal.

### PELVIC ABSCESS WITH GANGRENE OF THE VERMIFORM APPENDIX.

BY A. H. SCHENK, M. D., KENNEY, TEXAS.

[Read at the meeting of the Austin County Medical Association in Bellville, on July, 1893.]

PATIENT, A. Scharff, aged 28 years, a robust German, with a previous history of good health, came to me complaining of having had fever for two days. Without making a very careful examination, a diagnosis of remittent fever was made, and I prescribed accordingly, directing my patient, however, to report next day, if his fever had not subsided. Eight days later I was called to see him. He told me that my treatment had benefited him, but that he was not well. He was found still having fever, with a temperature of 103° F., a heavily coated tongue, dry and cracked; a pulse 120 per minute. His face bore an anxious expression; complexion was not clear. His mind was good after he was awake for a time, but just after being aroused from sleep, he would for a few moments stare around meaninglessly. His condition was very much that of a patient after a prolonged and constant elevation of temperature as often found in continued types of fever; though his peculiar countenance, tired look and muddy skin were strikingly characteristic of septic poisoning. For several days an expectant treatment was carried out without making a diagnosis, and in the meantime, on each visit a thorough examination was made. There was no pain in any region of the body, save a slight feeling of discomfort over the epigastrium; tympanites was rather marked. The rise and fall of his temperature was nearly regular, and indicated a continued fever, but his peculiar expression caused me to withhold a positive opinion.

On my fourth visit his condition, it was plain, was becoming more serious, as a progressive weakening was very apparent.

There were no sweats, no chilly sensations had occurred, and feeling the necessity of giving a positive opinion in regard to what the nature of the disease was, I felt justified in discarding the indication of septic fever and leaned towards continued fever, when my attention was attracted by the slightest difference between the R. and L. iliac regions. The sign could hardly be detected, and otherwise there was no reason to suspect pus in either of these regions. An exploratory puncture was made and pus found in the L. iliac region about an inch from the crest of the ilium. With the aid of Dr. J. C. McGregor, who gave chloroform, the abscess, which was extra-peritoneal, was opened.

By probing, the abscess cavity was found to be situated deep within the true pelvic cavity, the pus having burrowed its way to the surface between the pelvic wall and the peritoneum. By means of manipulation and the copious use of antiseptic water with a fountain syringe an enormous quantity of creamy pus, with shreds of dead tissue, were removed. An iodoformized gauze strip was inserted for further drainage, and after treatment consisted of irrigation with 1:3000 Hy Cl solution. The patient now did well: his temperature fell to 99° F.; pulse went down to between eighty and ninety per minute; his condition seemed to better itself daily, and I had discontinued my visits two days when I was sent for to come and see him in haste on the sixth day after opening the abscess. He had been suffering during the afternoon with intense colicky pains, and with these I found him. His temperature was 101° F., pulse 100 and upward per minute. On inquiry I learned that he had eaten a goodly portion of salt herring in the morning, and attributing his painful and worse state to this unwise taking of food, an anodyne was administered and a brisk dose of salts ordered to follow. On the following morning his condition was still painful, temperature 102½° F., pulse weak, 120 per minute, nausea marked and some retching, though his bowels had moved; abdomen was tender in general and disturbed, fingers were cold, entire surface clammy to the feel, and patient wore a very tired, hollow-eyed look. A well defined area of increased tenderness over the cæcum. Extreme prostration was evident to all, and his family readily consented to an exploratory incision. With the aid of three assistants I made an incision over the cæcum centrally to the area of increased tenderness, and finding the presenting coils of small intestines matted together, the opening was enlarged to about 4½ inches parallel with the linea alba. The adhesions were local, and after these had been, with slight effort, separated, the colon was traced to its blind end and the appendix found without difficulty. It was about two inches in length, of dark blue color, standing out against the right wall of the ilium, distended to its utmost with a murky mixture of offensive fluid and granular fecal matter. After dissecting it from a slight peritoneal attachment, the base was ligated with a double catgut ligature, and with small shreds of dead tissue adhering, removed. The stump looked well, was dressed with a hot $Hg\ O_2$ solution, and everything presenting a healthy appearance, with no oozing, the peritoneum was completely closed with one row of catgut sutures. The remaining layers of the ventral wall would, by preference, have been closed by two rows, but scarcity of suturing material, of necessity, compelled me to use only one row of interrupted sutures. After the operation the patient improved without interruption, though convalescence was tedious on account of the discharge of pus from the abscess in the left side, which did not cease in spite of the most thorough irrigation with various antiseptic fluids. After nearly two months time pus formations ceased and the abscess showed a tendency to get well under iodoformized glycerine injections. Now there is a remaining fissure probably two inches deep. The details of the clinical course of the case were given because these alone make it an unusual case, and show how difficult it was to find out what was the matter early in the case. It must be borne in mind that here in the outset was a severe case of septic fever that had lasted two weeks, and was jeopardizing the life of the patient, the real cause of which I had no clue to find out. There was no complaint of pain in the whole history of the case, primarily, nor any other symptom, objective or subjective, that pointed to the local sight of the trouble.

How important it is in such cases to evacuate pus early is known to us all; for the collection of pus in whatever portion of the body, and especially when in the region of a vital organ or cavity, must ever be regarded as a serious condition, and, as a rule, the early removal favors an early healing of the abscess cavity, preventing the formation of the increasing bulk of pus, which acts as a foreign body and besides is a dangerous source of septic poisoning as long as it remains in any cavity of the body, natural or unnatural, notwithstanding the knowledge of the fact that nature in so many instances shows wonderful powers of resistance to evil effects of its presence for various lengths of time. Pelvic abscess is treated very lightly in most text books of general surgery, and before concluding my paper I shall quote from Kelsey, to whose writings I am indebted for the clear understanding of the case herein reported. He says that an abscess with a very large collection of pus in the pelvi-rectal region gives rise to a continued rather than a high fever; that sweats and chills rarely occur and that pain generally is not marked. He speaks of bladder and rectum symptoms occurring. There was a little tendency to frequent urination in my case, but no more than sometimes found in fevers. Such an abscess has for its floor the lavator ani muscle, and in women is apt to open near the crest of the ilium; in men they generally burrow through the lavator ani muscle and open into the rectum or near the anus. The lavator ani muscle forms the floor of an abscess in this, the superior pelvi-rectal region. A probe, in my case, would of its own weight follow the sinus in this region, and the long tube of the syring was also insinuated to its whole length during irrigation. A positive diagnosis is made with difficulty by rectal and abdominal palpation. The gangrenous appendicitis in this case I believe developed independently of the abscess.

Appendicitis has recently claimed much attention in medical literature. To discuss its history and the many phases in which it shows itself would make a very long paper. Every leading journal has in the past year had articles on the subject. I desired only to give a practical report of this complicated case and its termination, which is recovery.

